# Response of cardiac pulse parameters in humans at various inclinations via 360° rotating platform for simulated microgravity perspective

**DOI:** 10.1038/s41526-023-00301-3

**Published:** 2023-07-18

**Authors:** Sagar Jagtap, Ajay Kumar, Bhoopesh Mahale, Jyotsana Dixit, Ashok E. Kalange, Rajesh Kanawade, Shashikala Gangal, Pandit Vidyasagar

**Affiliations:** 1 Department of Physics, Haribhai V. Desai College, Pune, MS 411002 India; 2 Physical and Materials Chemistry Division, CSIR-National Chemical Laboratory, Dr. Homi Bhabha Road, Pune, MS 411008 India; 3 Academy of Scientific and Innovative Research (AcSIR), Ghaziabad, 201002 India; 4 Department of Electronics, Savitribai Phule Pune University, Pune, MS 411007 India; 5 Department of Microbiology, Savitribai Phule Pune University, Pune, MS 411007 India; 6 Department of Physics, Tuljaram Chaturchand College, Baramati, Dist., Pune, 413102 MS India; 7 Department of Physics, Savitribai Phule Pune University, Pune, MS 411007 India

**Keywords:** Biophysics, Physiology

## Abstract

On the Earth, the human body is designed and adapted to function under uniform gravitational acceleration. However, exposure to microgravity or weightlessness as experienced by astronauts in space causes significant alterations in the functioning of the human cardiovascular system. Due to limitations in using real microgravity platforms, researchers opted for various ground-based microgravity analogs including head-down tilt (HDT) at fixed inclination. However, in the present study, an investigation of response of various cardiac parameters and their circulatory adaptation in 18 healthy male subjects was undertaken by using an indigenously developed 360° rotating platform. Cardiac pulse was recorded from 0° to 360° in steps of 30° inclination using piezoelectric pulse sensor (MLT1010) and associated cardiac parameters were analyzed. The results showed significant changes in the pulse shape while an interesting oscillating pattern was observed in associated cardiac parameters when rotated from 0° to 360°. The response of cardiac parameters became normal after returning to supine posture indicating the ability of the cardiovascular system to reversibly adapt to the postural changes. The observed changes in cardiac parameters at an inclination of 270°, in particular, were found to be comparable with spaceflight studies. Based on the obtained results and the proposed extended version of fluid redistribution mechanism, we herewith hypothesize that the rotation of a subject to head down tilt inclination (270°) along with other inclinations could represent a better microgravity analog for understanding the cumulative cardiac response of astronauts in space, particularly for short duration space missions.

## Introduction

Gravity is the only component of the Earth’s environment that has remained constant throughout the entire process of biological evolution^1^. Due to continuous exposure to normal gravity (1 g) conditions, great amounts of body fluid such as blood, move down into the lower part of the body since most of our daily activities are in upright or sitting postures. Around 75% of the total blood volume in a healthy person is found in the distensible veins i.e. in the lower part of the body. To maintain sufficient blood flow to the brain and oppose the Earth’s gravitational force, human cardiovascular system has evolved sophisticated mechanisms. For instance, most of the large and medium-sized veins and lymphatic vessels contain reinforced valves are closed to prevent the downward flow of blood and lymph^2^. However, in many situations, such as spaceflights, fighter planes and acrobatic flying, pilots, and astronauts experience rapid transitions between upright, sitting, and lying down postures. The change of posture may create an imbalance in the gravity perception similar to space and may lead to some physiological alterations.

Astronauts in space encounter much lower gravity than the Earth’s gravity, called microgravity or low gravity or simply µg. Transitioning from normal gravity environment (1 g) to microgravity, the body receives a different set of signals and tries to adapt to this new environment. It leads to changes in an astronaut’s circulatory system, which is originally accustomed to work against gravity on the Earth. In microgravity, the first and foremost physiological change caused due to the absence of downward gravitational pull is an accumulation of body fluid that occurs in the upper region, (µg induced cephalic fluid shift) leading to a puffy face syndrome^3^. Subsequently, a myriad of cardiovascular alterations such as orthostatic intolerance, pre-syncopal feelings due to postural stress, bone loss, muscle atrophy, decreased plasma volume and reduced exercise capacity are experienced by crew members during and/or after returning from space^3,4^. Vascular and cardiac dysfunction may be the cause for these effects. These effects become more severe with longer exposure to microgravity and require more lengthy recovery times after returning to the Earth. Even though short-duration spaceflights pose no major problems with post-flight functional and structural recovery, understanding of functional and physiological changes of the cardiovascular system in simulated microgravity are of great importance when planning for challenging extra vehicular activities and longer duration space missions.

Due to very limited access to human space missions and extreme difficulties in performing clinical experiments, the available statistical data on the cardiovascular system in space is unsystematic, inconsistent and restricted only to few cardiac parameters. In addition, several characteristics associated with the logistics of spaceflight presented significant limitations to the systematic study of human adaptation to microgravity^5^. Therefore, several Earth-based microgravity simulation techniques have been developed and used in the past few decades, which include head-down-tilt (HDT), dry/wet immersion, unilateral lower extremity limb suspension, supine bed rest etc. All these ground-based analogs have their own unique advantages and limitations in terms of applications to various physiological systems. The widely used analog for replicating the physiological effects of spaceflight on the Earth is the head-down tilting (HDT) bed rest, where the subject lies on a bed with head tilted down, usually 6° from the horizontal^6–10^. HDT establishes simulated microgravity environment based on body’s reaction to µ*g* conditions in terms of arterial pressure and fluid shifts similar to the ones experienced in weightlessness^11,12^. Although studies on HDT at fixed inclination provided comparable information on cardiovascular system in space, the net effect of microgravity on the body of an astronaut is the resultant of postures at different inclinations rather than at any fixed inclination, due to the absence of gravity. Therefore, in the present study, an attempt has been made to study the effects of different inclinations ranging from 0° to 360° on various cardiac parameters in healthy male subjects by attaching them to the 360° rotating platform.

## Results

### Pulse shape at different angles

**Fig. 1:**
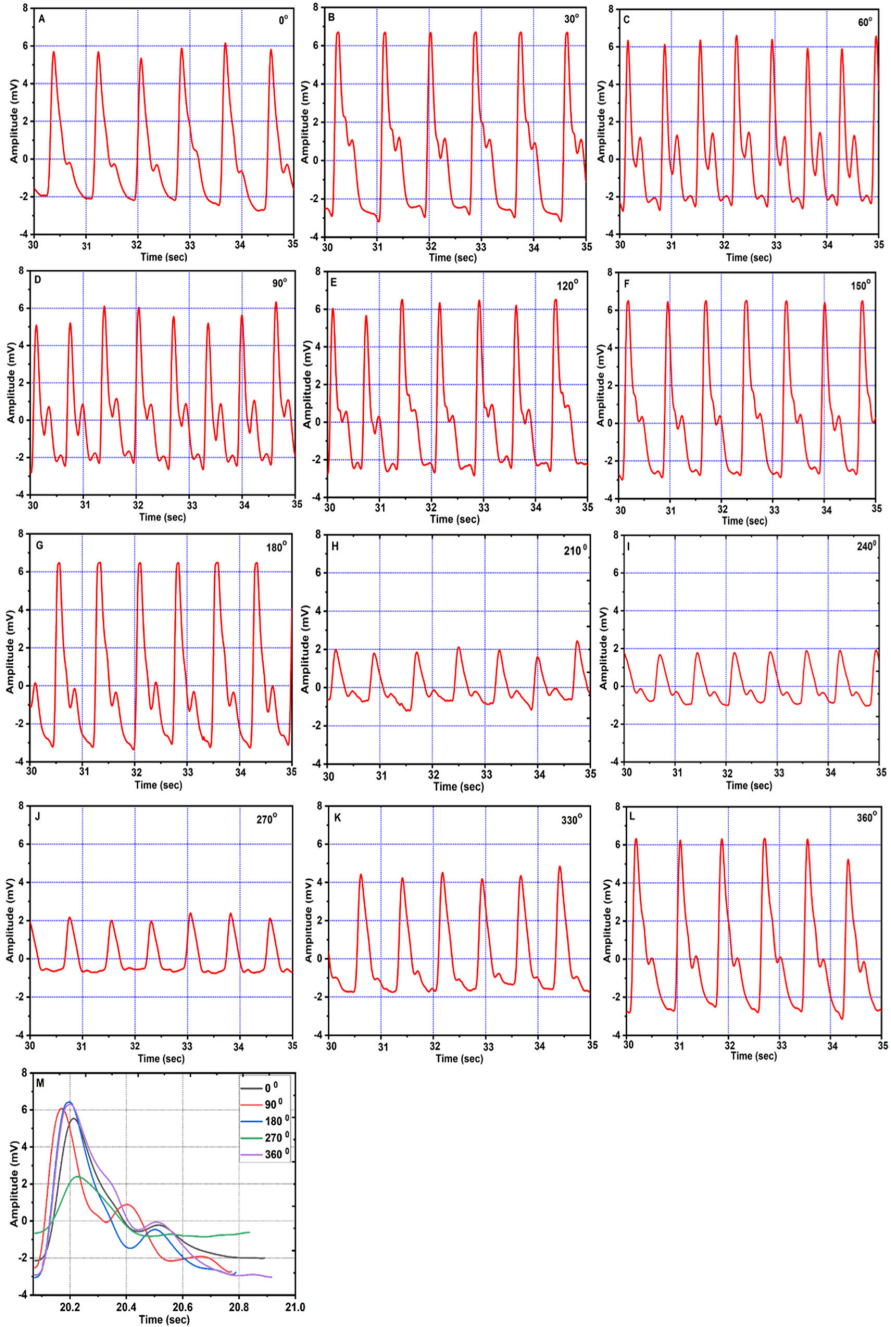
Arterial pulse wave shape for different inclinations. **A**–**L** Arterial pulse wave shapes for different inclinations ranging from 0° to 360° except 300°. **M** Comparative arterial pulse wave shapes for inclinations 0°, 90°, 180°, 270°, and 360°.

The arterial pulse wave amplitude (in mV) vs time (in sec) plots for the tilt angles from 0° to 360° (except 300°) in steps of 30°, are shown in Fig. 1. Changes were observed in pulse shape and, amplitude as well as its frequency. At supine (0°), the pulse shape showed normal behavior i.e., it had a gradual rise and a dicrotic notch on the falling slope. However, both systolic and diastolic peaks were found to be sharpened with increase in the frequency as well as amplitude when rotated from 30° to 90°. From a tilting angle of 120°, the amplitude of diastolic peak started decreasing which came to normal at an inclination 180°. After a 180°, again the amplitude of diastolic peak started decreasing, due to which, the dicrotic notch began to disappear from 210° and almost disappeared at 270°. From angle 330°, the dicrotic notch begins to appear and the pulse shape regained to its normal shape at 360°. The decrease in the intensity of systolic peak P1 was also observed for angles 240° to 270°, which started increasing from 330° and came to normal at 360°.

### Average t1, t2

**Fig. 2:**
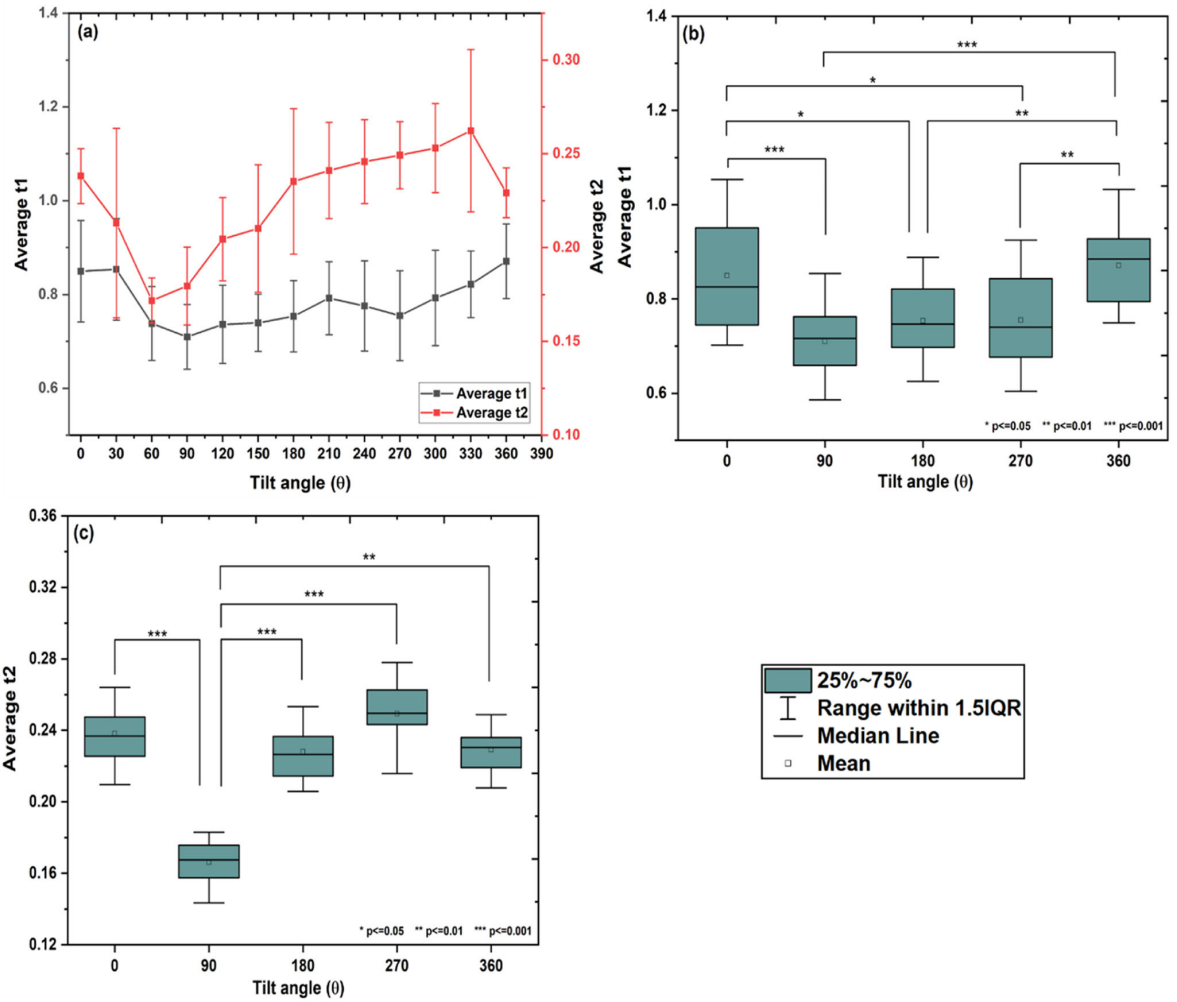
Variation in average t1 and t2 for different inclinations. **a** The average t1 (*n* = 18, mean ± SD) and average t2 (*n* = 18, mean ± SD) variations with change in tilt angle from 0° to 360°. The significant difference in average t1 and average t2 are observed with tilt group (one-way repeated measures of ANOVA, *p* < 0.05). Box plot for pair-wise comparison of (**b**) average t1 (*n* = 18, mean and 95% LCL-UCL: 0.870and 0.805–0.937, 0.715 and 0.674–0.756, 0.772 and 0.729–0.814, 0.751 and 0.692–0.811, and 0.870 and 0.823–0.918 for 0°, 90°, 180°, 270°, and 360° tilt positions; Tukey’s HSD multiple comparison post-hoc test, *p ≤ 0.05, **p ≤ 0.01 and ***p ≤ 0.001), and **c** average t2 (*n* = 18, mean and95% LCL-UCL: 0.235 and 0.227–0.244, 0.175 and 0.137–0.213, 0.229 and 0.213–0.245, 0.247 and 0.237–0.257, and 0.229 and 0.221–0.236 for 0°, 90°, 180°, 270°, and 360° tilt positions; Tukey’s HSD multiple comparison post-hoc test, *p ≤ 0.05, **p ≤ 0.01 and ***p ≤ 0.001) with the tilt group, respectively.

The variations in average values of t1 and t2 with change in the tilt angle from 0° to 360° are shown in Fig. 2a. A one-way repeated measure of ANOVA test was performed to access the difference in average values of t1 and t2 w.r.t. tilt group (0°, 90°, 180°, 270°, and 360° tilt positions). The significant difference in average values of t1 and t2 were observed with the tilt group (Mauchly’s sphericity test:*χ*^2^(9) = 8.06, *p* = 0.52 and *χ*^2^(9) = 44.49, *p* < 0.001; *F-* statistics: *F* (4,52) = 14.05, *p* < 0.001 and Greenhouse-Geisser correction: *F* (1.5, 20.3) = 9.17, *p* < 0.001 for average t1 and t2, respectively; one-way repeated measures of ANOVA, *p* < 0.05).

After confirmation of the statistical difference in average values of t1 and t2 with the tilt group from the one-way repeated measure of the ANOVA test, Tukey’s HSD multiple comparisons post-hoc tests were performed to identify the difference among the various combination of a tilt group. It was observed that, average t1 decreased up to 90° (*p* < 0.001), which remain unchanged up to 270° (*p* > 0.05), and then increased and came to normal at 360° (*p* < 0.01; Fig. 2b). Similarly, it was observed that average t2 decreased up to 90° (*p* < 0.001), after that increased, came to normal at 180° (*p* < 0.001), and then remain unchanged up to 360° (*p* > 0.05; Fig. 2c).

### Average P2/P1, t2/t1, and V/P1

**Fig. 3:**
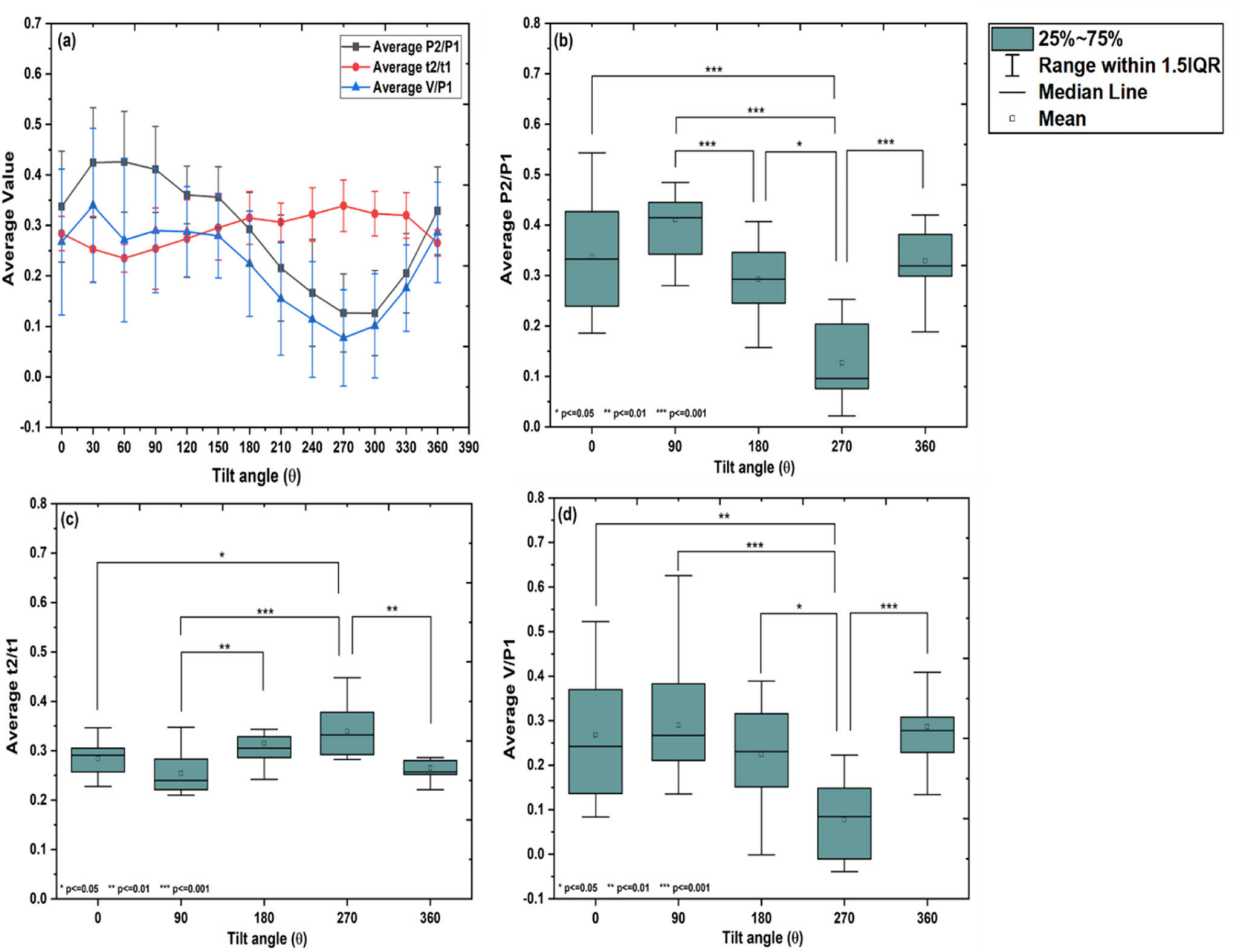
Changes in average P2/P1, average t2/t1 and average V/P1 for different inclinations. **a** The changes in average P2/P1 (*n* = 18, mean ± SD), average t2/t1 (*n* = 18, mean ± SD) and average V/P1 (*n* = 18, mean ± SD) with change in tilt angle from 0° to 360°. The significant difference in average P2/P1, average t2/t1 and average V/P1 are observed with tilt group (one-way repeated measures of ANOVA, *p* < 0.05). Box plot for pair-wise comparison of (**b**) average P2/P1 (*n* = 18, mean and95% LCL-UCL: 0.318 and 0.232–0.404, 0.373 and 0.321–0.424, 0.260 and 0.208–0.312, 0.126 and 0.071–0.181 and,0.320 and 0.252–0.388 for 0°, 90°, 180°, 27^00^, and 360° tilt positions; Tukey’s HSD multiple comparison post-hoc test, *p < =0.05, **p < =0.01 and ***p < =0.001), **c** average t2/t1 (*n* = 18, mean and 95% LCL-UCL: 0.285 and 0.264–0.306, 0.255 and 0.218–0.293, 0.229 and 0.281–0.317, 0.350 and 0.316–0.384, and 0.266 and 0.248–0.284 for 0°, 90°, 180°, 270°, and 360° tilt positions; Tukey’s HSD multiple comparison post-hoc test, *p ≤ 0.05, **p ≤ 0.01, and ***p ≤ 0.001), and average V/P1 (*n* = 18, mean and 95% LCL-UCL: 0.210 and 0.114–0.305, 0.234 and 0.148–0.319, 0.189 and 0.111–0.266, 0.086 and-0.015-0.189, and 0.237 and 0.177–0.297 for 0°, 90°, 180°, 270°, and 360° tilt positions; Tukey’s HSD multiple comparison post-hoc test, *p ≤ 0.05, **p ≤ 0.01 and ***p ≤ 0.001) with the tilt group, respectively.

The variation in average values of ratios P2/P1, t2/t1 and V/P1w.r.t. change in the tilt angle from 0° to 360° is shown in Fig. 3a. A significant difference in average values of P2/P1, t2/t1, and V/P1 was observed with tilt group (Mauchly’ssphericity test: *χ*^2^(9) = 12.12, *p* = 0.20, *χ*^2^(9) = 14.12, *p* = 0.11 and *χ*^2^(9) = 11.65, *p* = 0.23; *F*- statistics: *F*(4, 36) = 15.48, *p* < 0.001, *F*(4, 44) = 11.32, *p* < 0.001and *F*(4, 24) = 4.87, *p* = 0.005 for average P2/P1, t2/t1 and V/P1, respectively; one-way repeated measures of ANOVA, *p* < 0.05). From Tukey’s HSD multiple comparison post-hoc test, it was observed that, the average values of P2/P1 remain unchanged from 0° to 90° (*p* > 0.05), and then started decreasing from 90° to 180° (*p* < 0.001) till 270° (*p* < 0.05). From 270°, average values of P2/P1 started increasing and returned to normal at 360° (*p* < 0.001) (Fig. 3b). The values of average t2/t1 remain unchanged up to 90° (*p* > 0.05), then increased to 180° (*p* < 0.01), remain unchanged up to 270° (*p* > 0.05), and then decreased and came to normal at 360° (*p* < 0.01; Fig. 3c). The values of average V/P1 remain unchanged up to 180° (*p* > 0.05), decreased to 270° (*p* < 0.05), then increased and came to normal at 360° (*p* < 0.001; Fig. 3d).

### Blood pressure

**Fig. 4:**
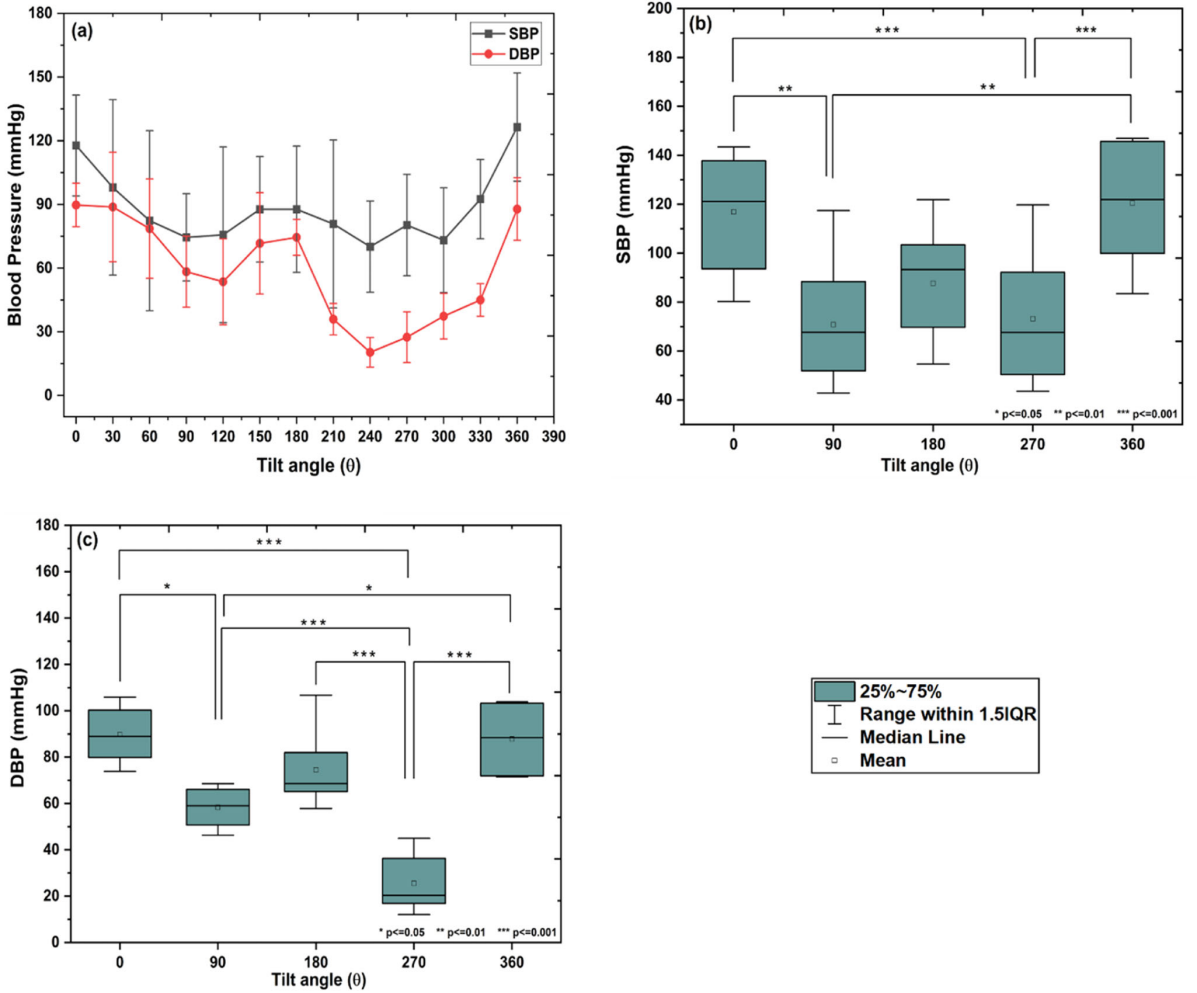
Changes in SBP and DBP w.r.t. different inclinations. **a** The changes in systolic blood pressure (SBP) (*n* = 18, mean ± SD) and diastolic blood pressure (DBP) (*n* = 18, mean ± SD) w.r.t. change in tilt angle from 0° to 360°. The significant difference in SBP and DBP are observed with tilt group (one-way repeated measures of ANOVA, *p* < 0.05). Box plot for pair-wise comparison of (**b**) SBP (*n* = 18, mean and 95% LCL-UCL: 117.77 and 102.28–133.26, 75.87 and61.41–90.33, 87.71 and 71.32–104.10, 80.26 and 61.05–99.47, and 126.41and 109.71–143.12 for 0°, 90°, 180°, 270°, and 360° tilt positions; Tukey’s HSD multiple comparison post-hoc test, *p ≤ 0.05, **p ≤ 0.01, and ***p ≤ 0.001), and **c** DBP (*n* = 18, mean and95% LCL-UCL: 89.71and79.60–99.82, 58.29 and 51.22–65.35, 74.48 and 60.48–88.48, 27.48 and 15.45–35.50, and 87.80 and 73.85–101.75for 0°, 90°, 180°, 270°, and 360° tilt positions; Tukey’s HSD multiple comparison post-hoc test, *p ≤ 0.05, **p ≤ 0.01 and ***p ≤ 0.001) with the tilt group, respectively.

The change in systolic blood pressure (SBP) and diastolic blood pressure (DBP) w.r.t. the change in the tilt angle from 0° to 360° is shown in Fig. 4a. A significant difference in means of systolic and diastolic blood pressure was observed with tilt group (Mauchly’ssphericity test: *χ*^2^(9) = 9.54, *p* = 0.38 and *χ*^2^(9) = 7.99, *p* = 0.53; *F*- statistics: *F*(4, 36) = 9.58, *p* < 0.001 and *F*(4, 36) = 26.20, *p* < 0.001 for systolic blood pressure and diastolic blood pressure, respectively; one-way repeated measures of ANOVA, *p* < 0.05). From Tukey’s HSD multiple comparison post-hoc test it was observed that, SBP decreased up to 90° (*p* < 0.01), remain unchanged up to 270° (*p* > 0.05), and then increased and came back to normal at 360° (*p* < 0.001; Fig. 4b). Similarly, DBP decreased up to 90° (*p* < 0.05), remain unchanged up to 180° (*p* > 0.05), then further decreased up to 270° (*p* < 0.001) and then increased and returned to normal value at 360° (*p* < 0.001; Fig. 4c).

### Heart rate

**Fig. 5:**
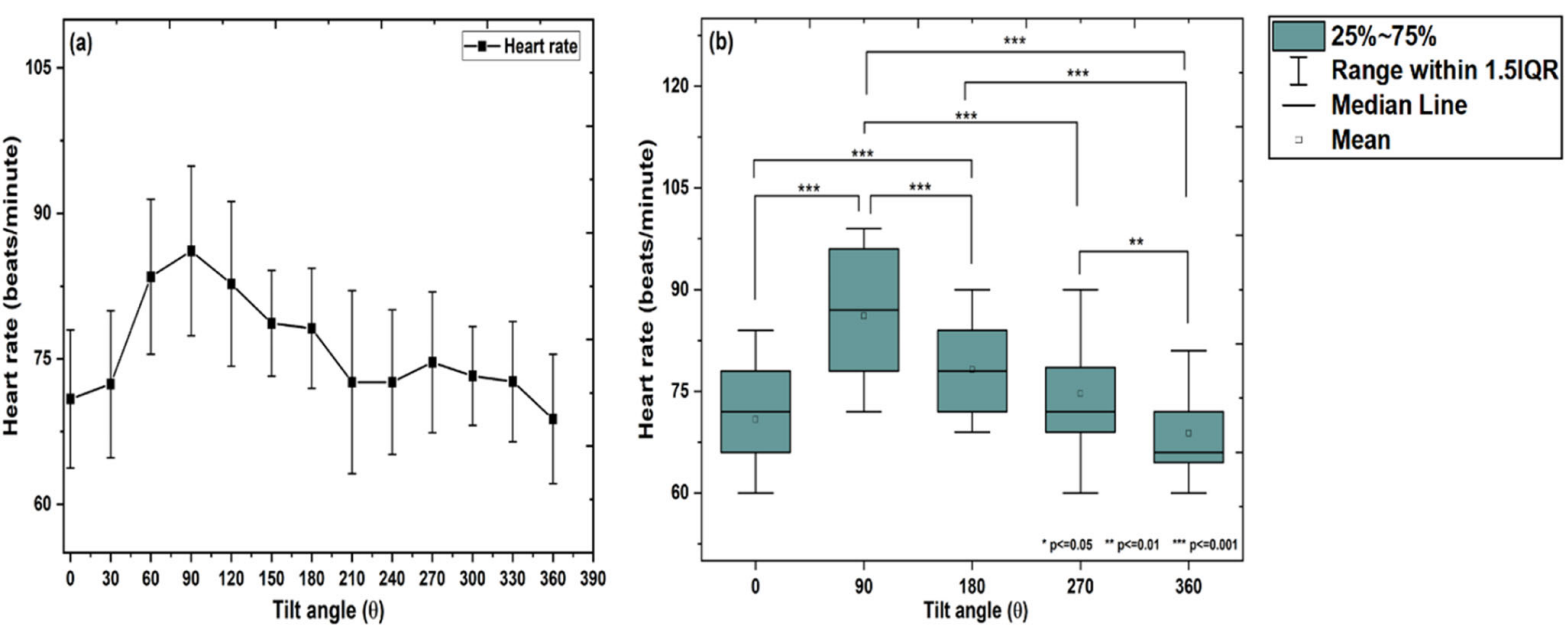
The variation in heart rate for different inclinations. **a** The changes in heart rate (HR) (*n* = 18, mean ± SD) with change in tilt angle from 0° to 360°. The significant difference in HR is observed with tilt group (one-way repeated measures of ANOVA, *p* < 0.05). **b** Box plot for pair-wise comparison of HR (*n* = 18, mean and 95% LCL-UCL: 71.9 and 69.74–74.10, 87.0 and 84.16–89.83, 78.7 and76.77–80.77, 74.6 and 72.32–76.97, and 69.2 and 67.03–71.41 for 0°, 90°, 180°, 270°, and 360° tilt positions; Tukey’s HSD multiple comparison post-hoc test, *p ≤ 0.05, **p ≤ 0.01 and ***p ≤ 0.001) with the tilt group, respectively.

Reciprocal of average values of t1 gives the heart rate of the pulse wave. At 0°, t1 was 0.845 sec i.e., the average length of one pulse wave is 0.845 s. Therefore, the number of pulse waves in 5 s record was 5/0.845 = 5.91, which gives the heart rate 71 beats/minute (5.91 × 12). The change in heart rate (HR) with the change in the tilt angle from 0° to 360° is shown in Fig. 5a. A significant difference in the means of HR was observed with tilt group (Mauchly’ssphericity test: *χ*^2^(9) = 14.47, *p* = 0.10; *F*- statistics: *F* (4, 156) = 49.66, *p* < 0.001; one-way repeated measures of ANOVA, *p* < 0.05). From Tukey’s HSD multiple comparisons post-hoc test it was observed that, HR increased up to 90° (*p* < 0.001), then decreased and back almost to normal at 180° (*p* < 0.001), remain unchanged upto 360° (*p* < 0.01; Fig. 5b).

### Stroke volume and cardiac output

**Fig. 6:**
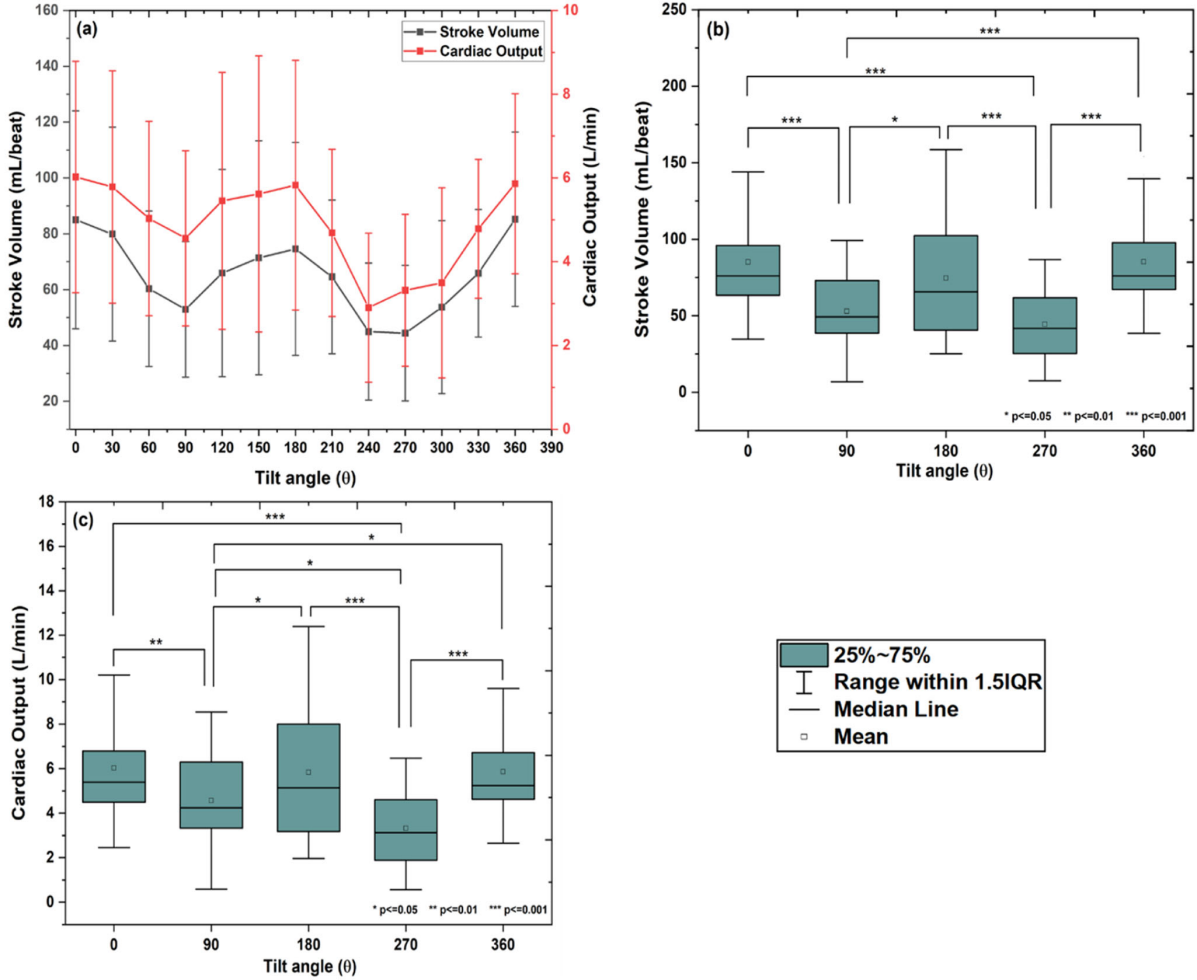
Changes in stroke volume and cardiac output for different inclinations. **a** The changes in stroke volume (*n* = 18, mean ± SD) and cardiac output (*n* = 18, mean ± SD) with change in tilt angle from 0° to 360°. The significant difference in stroke volume and cardiac output are observed with tilt group (one-way repeated measures of ANOVA, *p* < 0.05). Box plot for pair-wise comparison of (**b**) stroke volume (*n* = 18, mean and 95% LCL-UCL: 85.0 and 71.79–98.20, 52.9 and 44.71–61.15, 74.5 and 61.67–87.50, 44.4 and 36.20–52.64, and 85.2 and 74.64–95.78 for 0°, 90°, 180°, 270°, and 360° tilt positions; Tukey’s HSD multiple comparison post-hoc test, *p < =0.05, **p < =0.01 and ***p < =0.001), and **c** cardiac output (*n* = 18, mean and 95% LCL-UCL: 6.02 and 5.088–6.959, 4.55 and 3.851–5.267, 5.82 and 4.820–6.838, 3.31 and 2.702–3.929, and 5.86 and 5.135–6.589 for 0°, 90°, 180°, 270°, and 360° tilt positions; Tukey’s HSD multiple comparison post-hoc test, *p ≤ 0.05, **p ≤ 0.01, and ***p ≤ 0.001) with the tilt group, respectively.

Stroke volume was calculated from the area under the systolic curve of the arterial pulse waveform and the cardiac output was derived from the product of the stroke volume and heart rate. The change in stroke volume and cardiac output with the change in tilt angle from 0° to 360° is shown in Fig. 6a. A significant difference in means of stroke volume and cardiac output was observed with the tilt group (Mauchly’s sphericity test: *χ*^2^(9) = 41.93, *p* < 0.001 and *χ*^2^(9) = 40.37, *p* < 0.001; Greenhouse-Geisser correction: *F* (2.4, 87.1) = 14.55, *p* < 0.001 for stroke volume and cardiac output, respectively; one-way repeated measures of ANOVA, *p* < 0.05). From Tukey’s HSD multiple comparison post-hoc test, it was observed that, the stroke volume decreased up to 90° (*p* < 0.001), then increased up to 180° (*p* < 0.05), again decreased up to 270° (*p* < 0.001), and finally increased and returned to normal at 360° (*p* < 0.001; Fig. 6b). A similar trend was observed with the cardiac output, which firstly decreased up to 90° (*p* < 0.01), then increased up to 180° (*p* < 0.05), then again decreased up to 270° (*p* < 0.001), and then finally increased and returned to normal at 360° (*p* < 0.001; Fig. 6c).

## Discussion

In the present study, the changes in pulse shape and various cardiac parameters at different tilting angles (0^o^ to 360^o^) were analyzed using a rotating platform. As shown in Fig. 1, for supine position (0^o^), the pulse waveform showed a normal behavior. The amplitude (mV) of pulse waveform gradually increases, attains a maximum at P1 and falls down with a dicrotic notch on the falling slope. However, for higher inclinations 30° and 60°, significant alterations in the pulse shape were observed. The sharpening of both systolic as well as diastolic peaks and increase in the frequency at upright position (90°) indicates that the reflected wave is arriving faster when moving from supine to upright posture. This could be attributed to increased hydrostatic pressure in the arteries of the lower extremities and vice versa due to the postural change from supine to upright^13–15^. In addition to the arterial stiffness, the systolic wave depends mainly on the left ventricular ejection, whereas the diastolic wave is attributed to the reflections from the potential peripheral sites^16,17^. Also, it is documented that an increase in aortic stiffness can only increase aortic pulse pressure with a minor change in wave contour. In the present study, major changes in wave contour could be due to the changes in amplitude and timing of wave reflections as suggested by Nichols^18^. The decrease in the amplitudes of peak P1 and P2 after 180° inclination suggests an increase in pulse wave velocity for higher tilt angles, which leads to a faster arrival of the reflected wave. At angles from 210° to 270°, it seems that, due to the maximum fluid shift, pulse wave velocity increases, which causes a faster arrival of a reflected wave resulting in a drastic change in pulse shape. This leads to a decrease in the amplitude of both peaks and also the interval between the two peaks. As a result, the pulse wave lacks a dicrotic notch and is more rounded in shape especially at 270°^19^. In addition, some sort of tolerable anxiety/nervousness was observed in the subjects when rotated through inclinations from 210° to 270°. At 360 °, the pulse regains its normal shape which indicates that the effects on pulse shape are reversible. This could be attributed to quick adaptation of vascular tone and myogenic mechanism, as reported in the previous studies^20–23^. The variation in ratios of P2/P1, V/P1 and t2/t1 from 0° to 360° also support the observed change in pulse shape. The SBP and DBP both found to be decreased at an inclination up to 90°, increased again upto 180°, dropped to the minimum at 270°, and finally returned to normal at 360°. The significant finding of the present study indicates that the changes in cardiac parameters at an inclination 270° were on the similar lines with previous spaceflight and ground-based studies. For instance, out of total fifty-eight astronauts studied in space, forty-seven astronauts showed decrease in SBP and DBP as observed at 270° in our case^24^. The significant reduction in SBP and DBP was also observed in spaceflight studies performed 24 h before, during, and after spaceflight on Shuttle astronauts^25^. The similar decrease in blood pressure was observed in eight male cosmonauts (age 41–50 yrs, body-mass index of 22–28 kg/m^2^) during long-term missions (flight lengths of 162–196 days)^26^. After a 5-week head down tilt bed rest, SBP and pulse pressure were found to be decreased in ten young healthy volunteers^27^. Similar decrease in SBP and DBP were observed in twelve male subjects tilted from 45 head-up tilt to 45 head-down tilt on 3 separate days within a 2-week period^28^. The heart rate was found to be increased at upright (90°) however, no change in HR was observed at head down (270°) position. SV and CO showed a similar trend as that of SBP and DBP. Reduction in SV and CO is particularly evident in the upright posture^29–34^ or during lower body negative pressure^35,36^. These reductions are to a certain extent due to the reduced blood volume and cardiac atrophy^29,31,32,35,37–39^. The HR was found to be decreased or remain unchanged under short duration head-down tilt studies^28,40–42^ as compared to long duration^27,43^. Echocardiographic measurements obtained from short-duration space shuttle flights ranging from five to eight days in length showed an increase in heart rate and decrease in stroke volume before and after space flights^44,45^. These results are found to be consistent with the results obtained at 270° in the present study. Upon entering microgravity, the absence of the gravity vector decreases the hydrostatic pressure that induces the cephalic fluid shift resulting in increased cardiac output^19,46^. The increase in cardiac output is induced by increase in stroke volume as heart rate remain unchanged or decreased^47–49^. In the present study, the maximum headward fluid shift occurs at head down (270°) posture, while a decrease in SV was observed at 270°, which could be attributed to increase in pulse wave velocity. The similar decrease in stroke volume and peripheral vascular resistance was also observed during long-duration HDT bed rest and spaceflights to the ISS which could be the reason for ineffective maintenance of systemic arterial blood pressure^32,39,50–53^. Interestingly, decrease in stroke volume and cardiac output were observed mostly for long duration −6° head down tilt studies^27,42,43^. Echocardiographic data demonstrated that the lower stroke volume was associated with reduced cardiac size^44,54^. The variation in the results of cardiac parameters could be due to the duration of exposure to spaceflight or simulated microgravity and the experimental methodologies used. Furthermore, the cardiac pulse is a result of multi-parametric effect. In addition to many physiological processes related to cardiac cycle such as digestion, exercise; the brain-heart interaction, psychological effect may occur during rotation from 0° to 360°. This could probably explain the variations in the cardiac parameters reported in the present study. Based on the available data we propose that, 360° rotating platform could be more suitable analog for studying fast responsive physiological adaptations such as headward fluid shift, changes in blood pressure and blood velocity, stroke volume and cardiac output etc, as mostly observed in short duration spaceflight studies. At the same time, the HDT at any fixed inclination (mostly 6°) could provide a better analog for slow responsive physiological adaptations such as loss of bone, loss of muscle mass etc as observed from previous long duration spaceflight studies.

**Fig. 7:**
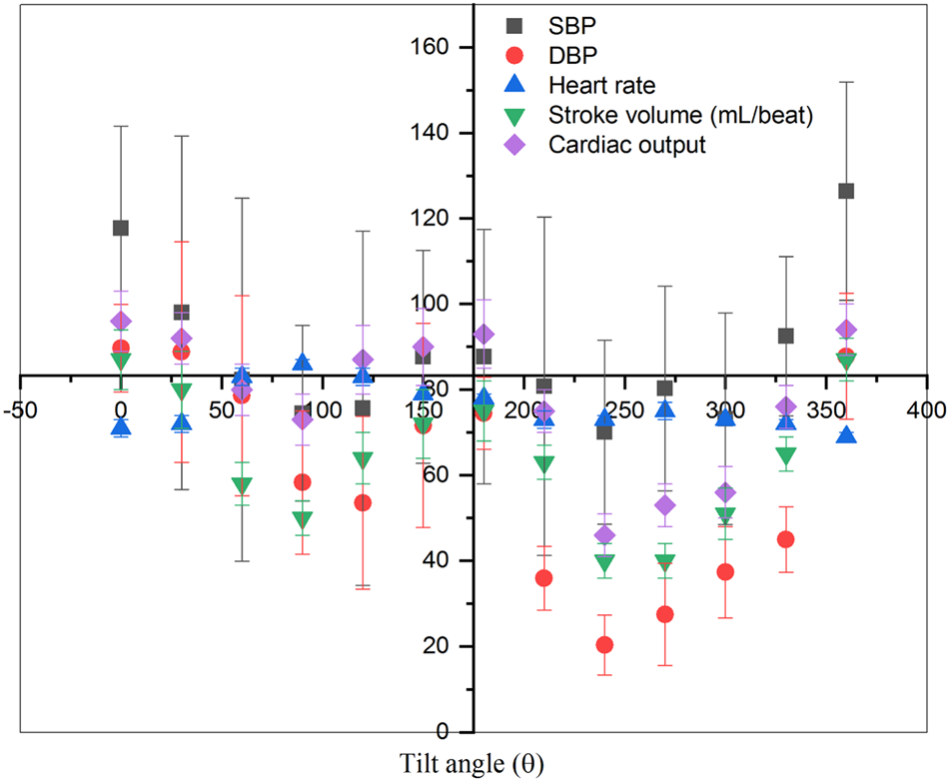
Collective response of various cardiac parameters for different inclinations. Collective response of various cardiac parameters SBP, DBP, heart rate, stroke volume, and cardiac output at various inclinations ranging from 0° to 360°.

Another important observation of this study is the interesting oscillating pattern of various cardiac parameters when rotated from 0° to 360° which is not reported earlier. Figure 7 shows the collective response (oscillatory pattern) of all cardiac parameters at various inclinations ranging from 0° to 360°. The reversibility of cardiac parameters at 360 ° through the transitions between the supine, upright, prone, and head-down postures show the ability of the cardiovascular system to reversibly adapt to the postural changes. This could be due to the baroreceptors, which play an important role in dealing with changes in blood pressure due to such postural changes through the process of baroreceptor reflex. However, exposure to real microgravity deconditions baroreceptor response^55,56^, or sometimes shows no significant change^57^. This kind of disagreement can be resolved by systematic planning of studies on cardiovascular parameters and baroreflex responses under different microgravity platforms.

In spaceflight or weightlessness conditions, the postural changes occur due to the absence of gravity, which covers the range of inclination from 0° to 360°. Although HDT studies at fixed inclination provided comparable results related to spaceflight experiments, cumulative response on an astronaut’s body may not be predicted with only certain specific inclination. Instead, it represents a model of unloading that is less confounded by in flight crew activities^58^. Unlike HDT, the 360° rotating platform provides a unique environment where subjects can be rotated from 0° to 360° with intermittent holding at different inclinations, a postural condition similar to space. However, it is imperative to be acquainted with the fluid redistribution mechanism in space and different terrestrial analogs in order to understand the cardiovascular response in space. On Earth, the human body experiences a uniform gravitational force of acceleration 9.8 m/s^2^. When a body in uniform gravitational force is opposed by a reaction force that occurs when standing on the ground, the gravitational force and reaction force exerted by the ground are in equilibrium. Complexity in the internal body structure does not allow the reaction force to spread uniformly but acts on each part of the body in a relative manner, which leads to the condition of the hydrostatic pressure gradient. The fluid redistribution on Earth is attributed to the residual hydrostatic pressure gradient developed in the arteries, veins, and interstitial fluids along the height of the body due to the gravitational field of the earth. Upon entering into space, the apparent gravitational force acting on a person becomes negligible, and the person experiences a condition of weightlessness. Initially, due to the hydrostatic pressure gradient, body fluid starts accumulating in the upper part of the body resulting into an expansion of the chest region, and the legs become slimmer. The fluid redistribution continues to refine throughout the body and retain its essential character of greatly increased fluid volume in the upper regions of the body at the expense of the lower regions. After a couple of weeks, this effect diminishes and the body reaches a homeostatic distribution of body fluid, which remains throughout their time in space. Thus, after spending some time in space, the hydrostatic pressure gradient becomes negligible and body fluid is uniformly distributed throughout the body.

**Fig. 8:**
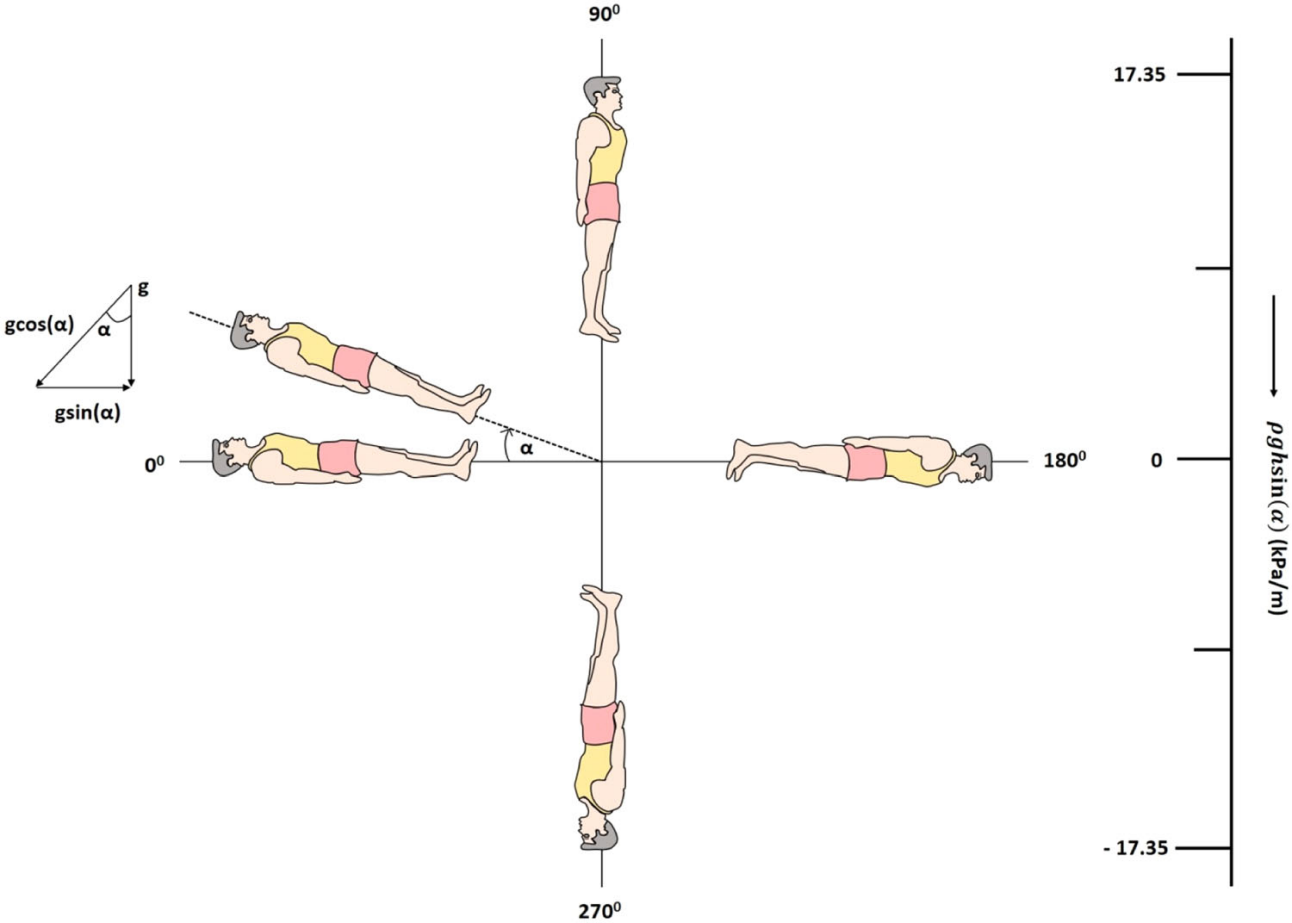
Schematic of an extended version of a human-shaped balloon model using 360° rotating platform. Schematic of an extended version of a human-shaped balloon model using 360° rotating platform which illustrates the nullification of hydrostatic pressure gradient, $\rho {gh}\,{\rm{sin }}\left(\alpha \right)$. The pressure gradient, $\rho {gh}\,{\rm{sin }}\left(\alpha \right)$ scale is given in terms of kPa/m on the right-hand side.

The most commonly used analog of fluid shift is HDT at some fix inclination say $\alpha$ (mostly a negative at - 6°) to promote headward fluid flow, a condition similar to space. In this case, the weight of the displaced fluid creates a hydrostatic force of magnitude $g\,{\rm{sin }}(\alpha )$ along the body axis so that the apparent hydrostatic pressure is ${\Delta P}_{h}=\rho {gh}\,{\rm{sin }}(\alpha )$ acting in the cephalic direction where $\rho$ is the density of fluid and $g$ is the gravitational acceleration. The perpendicular component $\rho {gh}\cos (\alpha )$ is also acting from the front to the back of the body. Though the physiological responses obtained with HDT are similar to that of space flight, HDT cannot remove the hydrostatic pressure gradient produced due to constant negative inclination. Instead, HDT could redirect the action of the hydrostatic pressure gradient which is well described by Nelson et al. (2014) using a hypothetical model of a human-shaped balloon filled with water^46^. The residue of hydrostatic pressure in this analog may impose a different loading environment or a biomechanical stress state in the body than would be seen in real microgravity where no hydrostatic pressure gradient is found after some time. In the present case of a 360° rotating platform, hydrostatic pressure gradient $\rho {gh}\,{\rm{sin }}(\alpha )$ is still acting on the body when rotated at various inclinations; however, after one complete rotation, the net or resultant effect of the hydrostatic pressure gradient is nullified due to vector addition. Thus, after returning to a supine position (360°), the hydrostatic pressure gradient no longer remains present, which results in the homeostatic fluid redistribution as experienced by an astronaut in space. This possibly explains the reversibility of cardiac parameters when a subject returns to the supine position. The similarities in the results at 270° and spaceflight studies are evident since, at this angle, the maximum fluid shift occurs at the upper part of the body, a postural condition similar to that during spaceflight. Thus, the technique of 360° rotation reported in the present study provides a simple and realistic case of both the fluid redistribution conditions as observed in space, viz., the initial non- homeostatic distribution of body fluid due to the presence of hydrostatic pressure gradient and homeostatic distribution due to its nullification after some time (around two weeks in space). Taking all quantities in SI units viz., average height of a person h = 1.77 m, density of water $\rho =1000\,{Kg}/{m}^{3}$, and acceleration due to gravity $=9.8\,m/{s}^{2}$, the hydrostatic pressure gradient for various inclinations is given in Table 1, calculated using the formula $\rho {gh}\,{\rm{sin }}\left(\alpha \right)$. Thus, the pressure gradient is near to zero at inclinations 0°, 180° and 360° while it appears maximum for 90° and 270°. For 90°, the pressure gradient is higher in the lower part of the body, while for 270°, it is higher for the upper part of the body. Therefore, when a subject has undergone a complete rotation, the net or resultant pressure gradient is averaged out to zero. The hypothetical model depicted in Fig. 8 illustrates the nullification of hydrostatic pressure gradient, $\rho {gh}\,{\rm{sin }}\left(\alpha \right)$ when a subject is rotated from 0° to 360°, which is an extended version of the human-shaped balloon model as described by Nelson et al. (2014).

**Table 1:** The hydrostatic pressure gradient $\rho {gh}\,{\rm{sin }}(\alpha )$ in KPascal/m for different inclinations ranging from 0° to 360° in steps of 30°.

Sr. No.	Inclination (degree)	Hydrostatic pressure gradient $(\rho {gh}\,{\rm{sin }}(\alpha ))$ (KPascal/m)
1.	0	0
2.	30	8.67
3.	60	15.02
4.	90	17.35
5.	120	15.02
6.	150	8.67
7.	180	0
8.	210	−8.67
9.	240	−15.02
10.	270	−17.35
11.	300	−15.021
12.	330	−8.67
13.	360	0

Based on the findings of the present study and the proposed extended version of the fluid redistribution mechanism, we herewith hypothesize that the head-down tilt posture at an inclination 270° along with other inclinations as discussed in this study could represent a better microgravity analog for understanding the cumulative cardiac response of astronauts in space, in particular short duration spaceflights. In this context, the approach used in the present study with different postures using a 360° rotating platform would give better insights into the response of astronaut’s body in space.

Though the results of the present study are promising and could be applied to understanding the effects of microgravity or spaceflight on the cardiovascular system, some limitations or precautionary measures should be taken into account. Firstly, as per the subject’s comfort, cardiac pulse was recorded from the wrist which is fixed straight down near the hip instead of equal height (altitude) with the heart. This kind of alignment may lead to a difference in the actual values of the cardiac parameters such as SBP and DBP in the aorta and SV and CO of the heart. However, it is documented that this difference has been found to be relatively small in healthy subjects and practitioners and is not commonly considered while interpreting the results^59,60^. In fact, such kind of difficulties in the measurement of cardiac parameters are expected in flight measurements as astronauts constantly change their positions and remain unbalanced during spaceflights.

The microgravity analog proposed in this study provides angle change in 2-Dimensions (2D) only but under microgravity, the subject moves in 3-Dimensions (3D); therefore, it is recommended that the experiments need to be performed on the 3D rotating platform for better interpretation of spaceflight results.

In summary, understanding the effects of microgravity, unavoidable physical factor in space, on cardiac pulse parameters is essential to venture upcoming short and long duration space expeditions. Limited accessibility of spaceflight missions promoted researchers to develop several ground-based analogs such as horizontal bed rest (HBR), head-down tilt bed rest (HDT), water immersion (WI), and dry water immersion (DWI) etc. The present study reported a new methodology to understand the human cardiovascular response in space using a 360° rotating platform and showed an alteration in the behavior of cardiac pulse shape with interesting oscillating pattern of cardiac parameters. The changes in various cardiac parameters at head down tilt posture 270° along with other inclinations could represent a better microgravity analog for understanding the cumulative cardiac response of astronauts in space, particularly for short duration space missions. Interestingly, the observed effects in pulse shape and other cardiac parameters were recovered soon after returning to the supine position indicating the adaptability of cardiovascular response to the simulated microgravity environment. The methodology used in the present study could be further explored in health assessment of astronauts, pilots, sky jumpers to overcome adverse effects by developing targeted countermeasures.

## Methods

### Subject selection

The experiments were performed on 18 healthy young male subjects in the age group 22 ± 2 years at Biophysics laboratory, Department of Physics, Savitribai Phule Pune University, Pune, MS, India. The average weight and height of the selected subjects were 60 Kg and 170 cm respectively. All subjects were non-smokers and were not on any medication. None of the subjects had a history of a cardiovascular or pulmonary disease. All subjects underwent complete medical examinations and did not reveal any pathological findings. They had blood pressure (125/84 ± 13/6) and heart rate (79 ± 10 beats/min) measured in sitting position before the actual experiment. Care was taken so that there was no discomfort to the subjects for breathing during the experiment. Before rotating, the subjects were kept at the supine position for about 5–10 min and pulse measurements were taken. All subjects gave their written informed consent and voluntarily participated in the study. The experiments were performed under a doctor’s supervision and followed the expert’s guidelines. All experimental details were explained to the subjects prior to the experiment.

### Experimental procedure

**Fig. 9:**
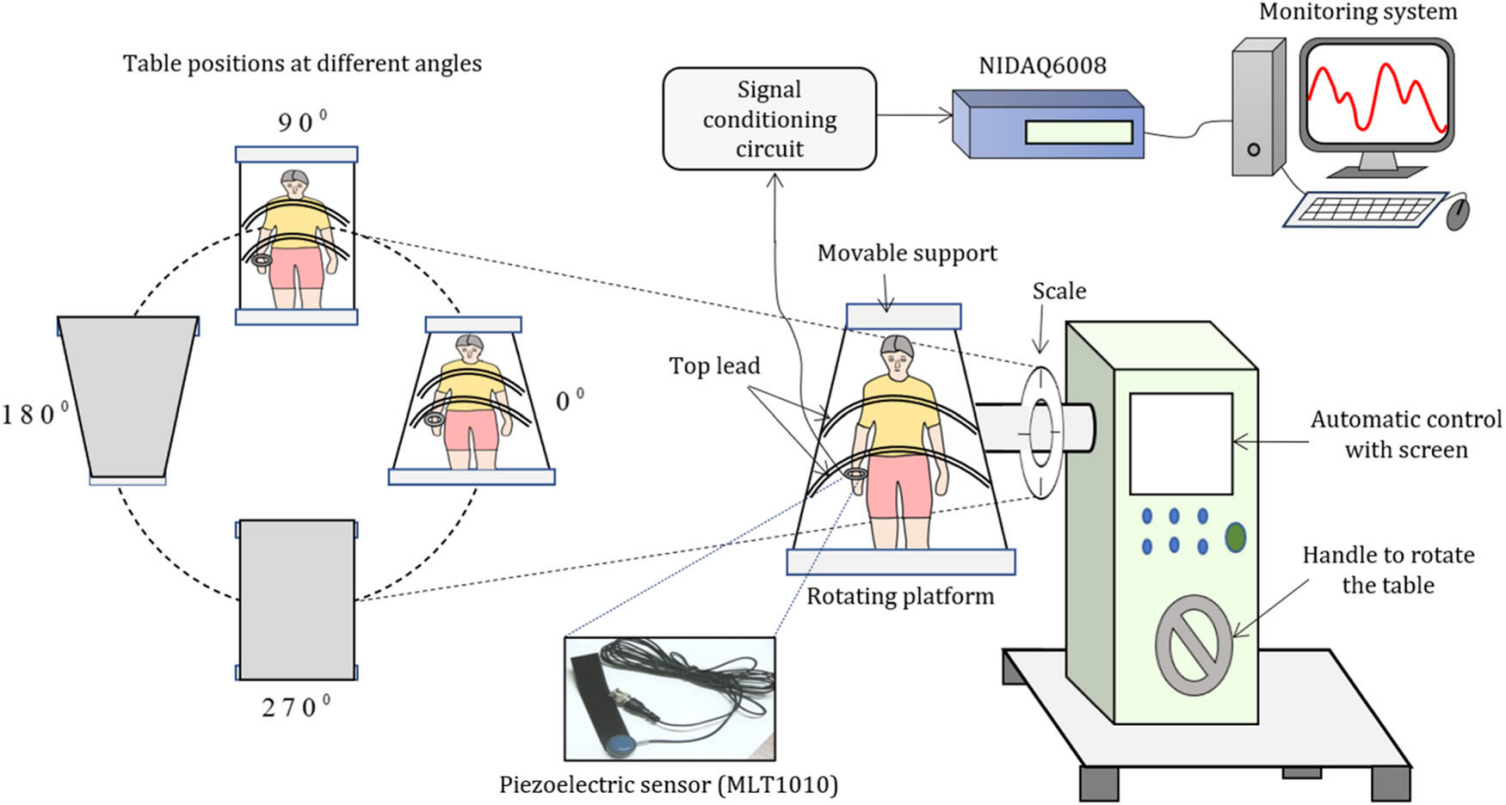
Experimental Set-up. Schematic of experimental set-up used for the arterial pulse measurement from subject’s wrist at different angles (0°–360°).

A rotating platform or a tilt table which can be rotated through 0°–360° in steps of 5°, 10°, and 15°, designed at the Biophysics laboratory, Savitribai Phule Pune University and developed by Hi-Q Electronics, Pune, India was used for these experiments. The schematic of the experimental setup used for the arterial pulse measurement from the subject’s wrist at different angles (0°–360°) is shown in Fig. 9. The tilt table consists of three main parts (i) a rotating platform, (ii) movable cotton support for height adjustment and iii) a top lead which keeps the subjects attached to the platform comfortably during rotation. For the experiment, the top lead was opened and the subject was attached to a rotating platform with the help of cotton belts. Then the top lead was closed and tightened to keep the subject firmly attached to the platform during rotation. Rotation speed was kept 1 rpm to avoid jerks and to maintain the subject’s comfort. The same procedure was repeated for all eighteen subjects. The piezoelectric pulse sensor (MLT1010, AD Instruments), size (diameter × thickness): 22 × 12 mm (0.87″ x 0.47″), cable length: 1 m (3.3′), typical output: 50–200 mV, frequency response: 2.5–5000 Hz, shown in Fig. 9 was used to detect human pulse waveform. The sensor was placed on the wrist using a Velcro^®^ strap. The area of interest on the wrist was cleaned using alcohol and the piezoelectric sensor was mounted using a rubber band.

The sensor converts the wrist pulse pressure into an electrical signal, which further passes through the signal conditioning unit and NI DAQ-6008 data acquisition unit. Subjects were rotated in a clockwise direction through the steps of 30° with the precision of ±2° and the pulse was recorded for 60 s. Each position was maintained for 3 min for pulse measurement and corresponding data was acquired. Due to movements or irregular breathing, anomalous pulses were rejected from the analysis. The Research Ethics Committee, Savitribai Phule Pune University, Pune, MS, India approved this study.

### Signal conditioning unit

**Fig. 10:**
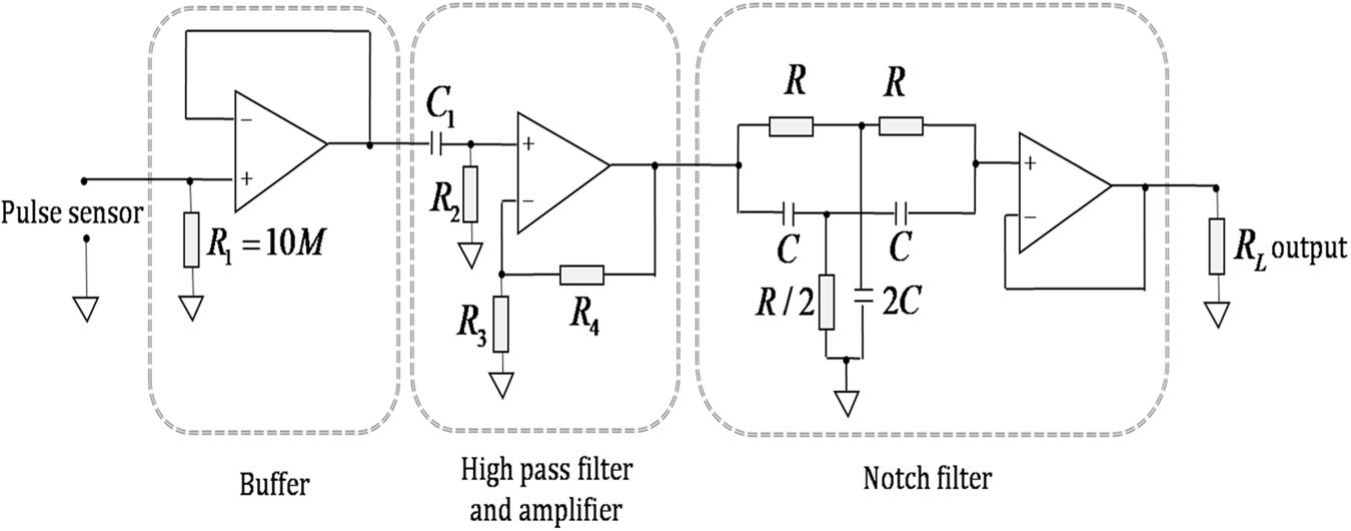
Signal conditioning unit. Circuit diagram of signal conditioning unit.

The circuit diagram of the signal conditioning unit, used in the present application, is shown in Fig. 10. Considering the high output impedance of the piezoelectric pulse sensor, a buffer is designed as the first stage of the circuit. Small output of the sensor is amplified using non-inverting operational amplifier. Respiration frequency of the human (0.3–0.4 Hz) as well as 50 Hz mains noise interfering with the human arterial pulse is filtered out using high pass filter and a notch filter respectively. A12-bit data acquisition card NI-DAQ 6008 is used to acquire the data in PC for further processing. A lab-view code (VI) is developed to acquire display and store the pulse data and is used to acquire arterial pulse wave signal. Average values of t_1_, t_2_, ratios P_2_/P_1_, t_2_/t_1_, V/P_1_, and systolic blood pressure (SBP), diastolic blood pressure (DBP), heart rate (HR), stroke volume (SV), and cardiac output (CO) were calculated from arterial pulse wave signal by using Origin Pro 2020 software.

### Pulse waveform analysis

**Fig. 11:**
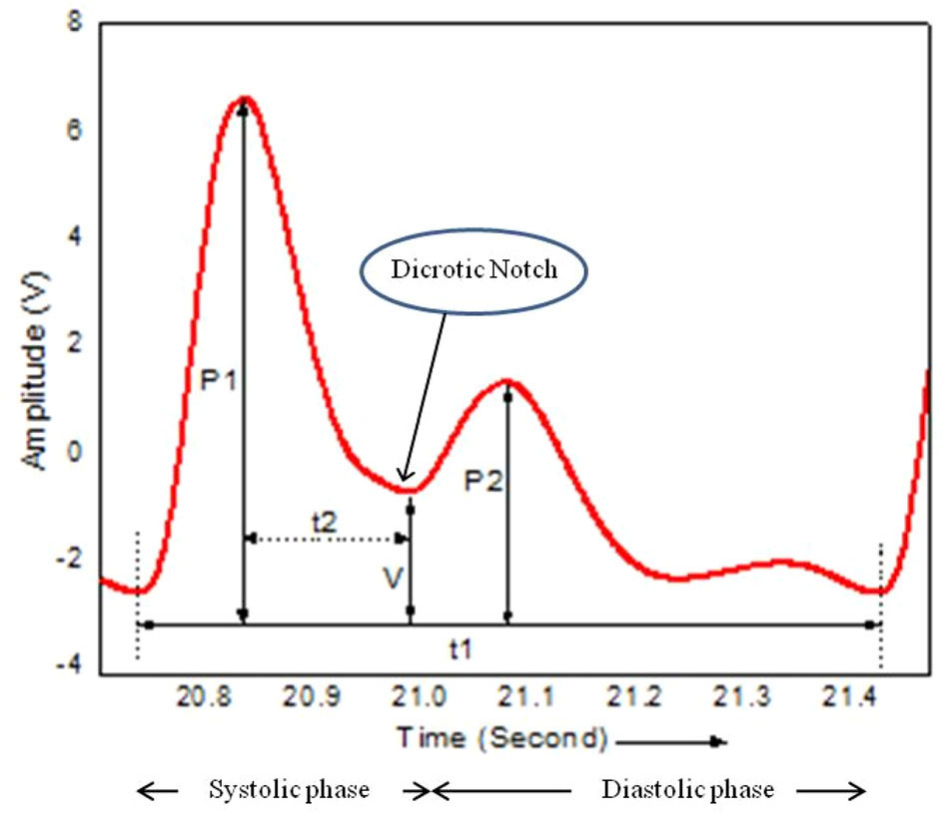
A typical arterial pulse waveform. A typical arterial pulse waveform showing systolic and diastolic phases.

Pulse waveform analysis (PWA) is a simple, non-invasive, and convenient technique, which relates many important factors such as arterial blood pressure, vascular resistance, and cardiac output, and can be regarded as the best clinical indicator to diagnose cardiovascular diseases. It is a robust and reproducible method among the non-invasive methods of evaluating cardiovascular parameters and provides vital information about the index of arterial compliance and stiffness^18,61–63^. In cardiac cycle, the arterial blood pressure raises and falls in a pattern corresponding to the phases of the cycles of the heart. Figure 11 shows a typical arterial pulse waveform. The cardiac pulse waveform is generally divided into two parts (i) forward traveling wave or systolic phase, (ii) reflected wave returning from the periphery or diastolic phase; and the junction of which is called the dicrotic notch.

In the systolic phase, the ventricles contract (ventricular systole), their walls squeeze the blood inside their chambers and it is forced into the pulmonary artery and aorta. As a result, the pressures in these arteries rise sharply. The maximum pressure achieved during such a ventricular contraction is the systolic pressure (P1). In the diastolic phase, the ventricles relax (ventricular diastole), and begin to fill with blood again to prepare for the next contraction and the arterial pressure drops. The lowest pressure that remains in the arteries before the next ventricular contraction occurs is termed as diastolic pressure (P2). Ideally, for a young person whose arterial tree is generally soft and compliant, the reflected wave coincides with the diastole phase of the cardiac cycle, which consequently leads to the absence of pre-dicrotic wave^18,63^. The parameter t1 represent the time required to complete one cardiac cycle and used to calculate the heart rate, while t2 represents the time between the maximum systolic pressure peak (P1) and the valley (V). The values of P1, P2, t1, t2, and valley (V) are used as marks for signal analysis and comparison.

### Statistical analysis

The statistical analysis was performed for the cardiac pulse parameters of total 18 healthy male subjects (*n* = 18) who had completed the 360° rotation experiment without any major discomfort or pain. To compare the mean of the pulse parameters (t1, t2, t2/t1, P2/P1, V/P1), SBP, DBP, HR, SV, and CO, a tilt group of five different tilt positions such as 0°, 90°, 180°, 270°, and 360° was formed. A one-way repeated measure of ANOVA test was used to access the differences in the mean for the tilt group. Whenever differences were detected, Tukey’s HSD (Honestly Significant Difference) multiple comparison post-hoc test was performed to identify the differences among the various combinations of tilt group. All these statistical tests were performed after eliminating outliers using 1.5 IQR (interquartile range) from the data. In all statistical tests, a “*p* value” <0.05 was considered statistically significant. The data of the subjects who experience pain/fear and felt uncomfortable (may be due to the orthostatic intolerance) has not been considered in the result analysis.

### Reporting summary

Further information on research design is available in the Nature Research Reporting Summary linked to this article.

## Supplementary information

Reporting Summary

## Data Availability

The data generated during or analyzed during this study are available from the authors upon reasonable request.
