# Supplementary material for: Response of cardiac pulse parameters in humans at various inclinations via 360° rotating platform for simulated microgravity perspective
Source: NPJ Microgravity. 2023 Jul 18;9:54. doi: 10.1038/s41526-023-00301-3 (PMC10354073; doi:10.1038/s41526-023-00301-3)
Supplement: Supplementary file 1 — Reporting Summary [file 41526_2023_301_MOESM1_ESM.pdf]

nature portfolio

Corresponding author(s): DBPR: NPJMGRAV-00892R

Last updated by author(s): Mar 24, 2023

Reporting Summary

Nature Portfolio wishes to improve the reproducibility of the work that we publish. This form provides structure for consistency and transparency in reporting. For further information on Nature Portfolio policies, see our Editorial Policies and the Editorial Policy Checklist.

Statistics

For all statistical analyses, confirm that the following items are present in the figure legend, table legend, main text, or Methods section.

n/a	Confirmed	
☐	☒	The exact sample size (*n*) for each experimental group/condition, given as a discrete number and unit of measurement
☐	☒	A statement on whether measurements were taken from distinct samples or whether the same sample was measured repeatedly
☐	☒	The statistical test(s) used AND whether they are one- or two-sided<br>*Only common tests should be described solely by name; describe more complex techniques in the Methods section.*
☒	☐	A description of all covariates tested
☒	☐	A description of any assumptions or corrections, such as tests of normality and adjustment for multiple comparisons
☐	☒	A full description of the statistical parameters including central tendency (e.g. means) or other basic estimates (e.g. regression coefficient) AND variation (e.g. standard deviation) or associated estimates of uncertainty (e.g. confidence intervals)
☐	☒	For null hypothesis testing, the test statistic (e.g. *F*, *t*, *r*) with confidence intervals, effect sizes, degrees of freedom and *P* value noted<br>*Give P values as exact values whenever suitable.*
☒	☐	For Bayesian analysis, information on the choice of priors and Markov chain Monte Carlo settings
☒	☐	For hierarchical and complex designs, identification of the appropriate level for tests and full reporting of outcomes
☒	☐	Estimates of effect sizes (e.g. Cohen's *d*, Pearson's *r*), indicating how they were calculated

Our web collection on statistics for biologists contains articles on many of the points above.

Software and code

Policy information about availability of computer code

Data collection	No software was used
Data analysis	No software was used

For manuscripts utilizing custom algorithms or software that are central to the research but not yet described in published literature, software must be made available to editors and reviewers. We strongly encourage code deposition in a community repository (e.g. GitHub). See the Nature Portfolio guidelines for submitting code & software for further information.

Data

Policy information about availability of data

All manuscripts must include a data availability statement. This statement should provide the following information, where applicable:

- Accession codes, unique identifiers, or web links for publicly available datasets
- A description of any restrictions on data availability
- For clinical datasets or third party data, please ensure that the statement adheres to our policy

The data generated during or analyzed during this study are available from the authors upon reasonable request.

Human research participants

Policy information about studies involving human research participants and Sex and Gender in Research.

Reporting on sex and gender	No sex- and gender -based analyses were performed
Population characteristics	See above
Recruitment	All subjects were non-smokers and were not on any medication. None of the subjects had a history of a cardiovascular or pulmonary disease. All subjects underwent complete medical examination and did not reveal any pathological findings. All subjects gave their written informed consent and voluntarily participated in the study. The experiments were performed under a doctor's supervision and followed the expert's guidelines.
Ethics oversight	This study was approved by the Research Ethics Committee of Savitribai Phule Pune University, Pune, MS, India.

Note that full information on the approval of the study protocol must also be provided in the manuscript.

Field-specific reporting

Please select the one below that is the best fit for your research. If you are not sure, read the appropriate sections before making your selection.

☒ Life sciences ☐ Behavioural & social sciences ☐ Ecological, evolutionary & environmental sciences

For a reference copy of the document with all sections, see nature.com/documents/nr-reporting-summary-flat.pdf

Life sciences study design

All studies must disclose on these points even when the disclosure is negative.

Sample size	Sample sizes were chosen arbitrarily
Data exclusions	Subjects were not excluded but few subjects who failed to complete the experiment due to uneasiness were discontinued and their data has not been added in the results as the data set was incomplete. Total 18 subjects completed the rotation so the data of all these 18 subjects were recorded and analyzed in the present study.
Replication	After completing one rotation, the body is likely to get conditioned. The subsequent rotation/s of the same subject may deviate the actual effect caused due to one rotation. So, instead of repeating the rotation on the same subject, a group of subjects (age group 22 ± 2 years) were preferred.
Randomization	Subject from the age group (22 ± 2 years) were randomly selected for the experiment.
Blinding	All experimental details were explained to the subjects prior to the experiment. All subjects gave their written informed consent and voluntarily participated in the study. The experiments were performed under a doctor's supervision and followed the expert's guidelines.

Reporting for specific materials, systems and methods

We require information from authors about some types of materials, experimental systems and methods used in many studies. Here, indicate whether each material, system or method listed is relevant to your study. If you are not sure if a list item applies to your research, read the appropriate section before selecting a response.

Materials & experimental systems

n/a	Involved in the study	
☒	☐	Antibodies
☒	☐	Eukaryotic cell lines
☒	☐	Palaeontology and archaeology
☒	☐	Animals and other organisms
☒	☐	Clinical data
☒	☐	Dual use research of concern

Methods

n/a	Involved in the study	
☒	☐	ChIP-seq
☒	☐	Flow cytometry
☒	☐	MRI-based neuroimaging
